# Mathematical modelling of expanded bed adsorption – a perspective on in silico process design

**DOI:** 10.1002/jctb.5595

**Published:** 2018-04-06

**Authors:** Victor Koppejan, Guilherme Ferreira, Dong‐Qiang Lin, Marcel Ottens

**Affiliations:** ^1^ Delft University of Technology Department of Biotechnology, Van der Maasweg 9, 2629 HZ Delft The Netherlands; ^2^ DSM Biotechnology Center Center of Integrated BioProcessing, Alexander Fleminglaan 1 2613 AX Delft The Netherlands; ^3^ College of Chemical and Biological Engineering Zhejiang University Hangzhou China

**Keywords:** Bioseparations, Chromatography, Mathematical Modelling, Process Development, Downstream

## Abstract

Expanded bed adsorption (EBA) emerged in the early 1990s in an attempt to integrate the clarification, capture and initial product concentration/purification process. Several mathematical models have been put forward to describe its operation. However, none of the models developed specifically for EBA allows simultaneous prediction of bed hydrodynamics, mass transfer/adsorption and (unwanted) interactions and fouling. This currently limits the development and early optimization of EBA‐based separation processes. In multiphase reactor engineering, the use of multiphase computational fluid dynamics has been shown to improve fundamental understanding of fluidized beds. To advance EBA technology, a combination of particle, equipment and process scale models should be used. By employing a cascade of multiscale simulations, the various challenges EBA currently faces can be addressed. This allows for optimal design and selection of equipment, materials and process conditions, and reduces risks and development times of downstream processes involving EBA. © 2018 The Authors. *Journal of Chemical Technology & Biotechnology* published by John Wiley & Sons Ltd on behalf of Society of Chemical Industry.

## INTRODUCTION

**Figure 1:**
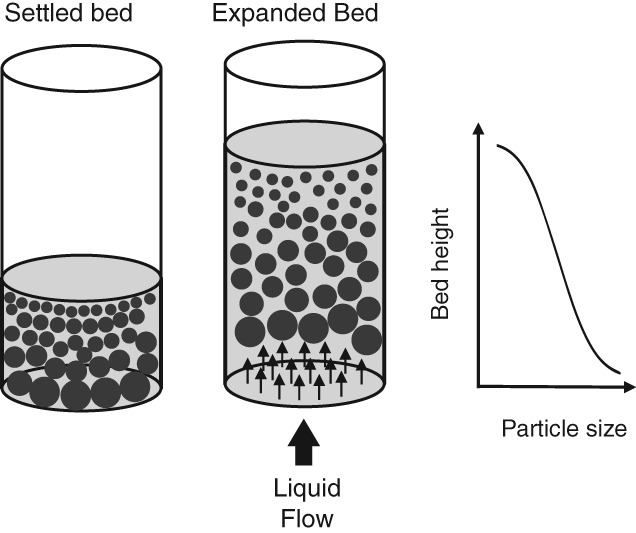
Schematic overview of bed expansion. A settled bed is expanded by applying an upwards fluid flow. Once the bed is fully expanded and has reached an equilibrium state, it develops a gradient in particle size and density, which reduces particle back mixing.

The downstream processing (DSP) of biological products typically involves a sequence of unit operations to remove biomass, capture the target, purify and finally formulate it.^1^ The combined impact of multiple unit operations and incomplete recoveries at each step translates to reduced overall yields and makes DSP of bioproducts very costly.^2^, ^3^ Reducing the number of steps in DSP would therefore be beneficial to the overall process economics. In EBA the bed of adsorbent is mildly fluidized (expanded) and classified by applying an upward liquid flow (shown schematically in Fig. 1). This allows cells and debris to move through the bed relatively unhindered while the desired product is captured by means of adsorption. The technology of EBA is attractive because it fuses three unit operations (clarification, capture, concentration and partial purification) into one, promising improved overall yield.^4^

EBA came to life in the early 1990s to combine and replace clarification, capture and initial product concentration/purification.^5^ Although there are examples of the use of EBA for large‐scale separations,^4^, ^6^, ^7^ the technology has not been widely adopted in industry.^8^ To successfully apply EBA in an industrial setting, operating conditions should be optimized early in the process development. For traditional packed bed chromatography (PBC), the use of mechanistic models has improved the design and optimization of both equipment and processes.^8^, ^9^ Simulation of EBA has the added difficulty of a non‐stationary solid phase and the application of an unprocessed fermentation broth or cell suspension. Therefore, mechanistic modelling of EBA systems has not reached the same level of maturity as in PBC. In many industrial applications, the challenge still remains in dealing with unwanted interactions between the resin and broth components such as biomass, lipids or DNA. These interactions result in resin fouling and particle agglomeration, which in turn may cause collapse of the bed.^10^ Improvements in controlling biomass adhesion have been made by modifying the particle surface^11^, ^12^, ^13^ and the fluid phase pH or ionic strength.^14^ So far, these techniques have not yet been included or combined with available predictive models for bed hydrodynamics. The aim of this paper is to provide an overview of currently available mechanistic models for EBA systems, extend this with a summary of models for liquid‐solid fluidized beds (LSFBs) and propose how these models can be extended to allow for in silico optimization of EBA systems. This paper is organized as follows: (i) mathematical models describing EBA behaviour with specific focus on DSP in bioprocessing; (ii) stability analysis and multiphase computational fluid dynamics (CFD) for LFSBs; (iii) a case study which will demonstrate how CFD can be used to evaluate designs of fluid distributors for EBA columns; (iv) current challenges and potential improvements for modelling and simulation of EBA systems; and (v) summary and conclusions.

## REVIEW OF MATHEMATICAL MODELS DESCRIBING EBA BEHAVIOUR

Modelling and simulation of PBC has been shown highly beneficial for equipment and process development. In addition to the transfer and adsorption phenomena, modelling EBA presents additional challenges arising from axial distribution of particle properties (size, density, volume fraction) and unwanted interactions between adsorbent particles and biomass. Throughout the history of EBA a number of mathematical models has been proposed for describing EBA intended for application in bio‐separation processes. These models, categorized as ideal steady state, ideal dynamic and non‐ideal bed expansion, are reviewed in the coming sections.

### Ideal steady state bed expansion

For packed bed chromatography, the column efficiency can be described by the ‘height equivalent to a theoretical plate’ (HETP) model, which is based on the ‘tanks in series’ model by Levenspiel.^15^ The HETP model assumes that all hydrodynamic effects in the column are derived from the evolution of an inert tracer pulse, described by a Gaussian distribution function. The number of tanks in series, or plate number (N), can be calculated as the squared ratio of the retention time (t_m_) and the peak variance (σ), N = (t_m_/σ)^2^. The height of a single plate can then be calculated as HETP = L/N, where L is the column length. Several authors^16^, ^17^, ^18^, ^19^, ^20^ have extended this model to EBA columns. The bed expansion and voidage was approximated by assuming a constant particle size, and applying the Richardson‐Zaki Equation^21^ to account for the hindered settling velocity. Pällson and co‐workers^16^ and Theodossiou et al. 18 report that a combination of the Richardson‐Zaki and HETP models provides misleading information about the plate number and the axial dispersion in the column. To allow comparison of different resins and columns, Pällson and co‐workers^16^ proposed to base the plate number and vessel dispersion number on the settled bed height, but did not include a particle size distribution.^17^ In addition, Hubbuch et al. 22 pointed out that the system's piping, the flow distributor and column outlet design all contribute to the vessel dispersion number and the HETP. This further illustrates that using only the HETP and dispersion values offers limited insight into the physical behaviour of expanded beds.

The axial‐dispersion model, widely used to describe PBC performance,^23^, ^24^ has been employed by several authors to describe EBA columns, whose work will be summarized in this section. The simplest of PBC models assumes a constant particle size and void fraction along the bed height, after which the governing transport equation ((1)) becomes the same as that for a PBC model^25^

(1) $$\frac{\partial c}{\partial t}+\frac{u_{f}}{\varepsilon_{f}}\frac{\partial c}{\partial x}=D_{f}\frac{\partial^{2}c}{\partial x^{2}}-S$$

Here, c is the solute concentration, u_f_ is the liquid velocity, ε_f_ is the liquid fraction and S is the source/sink term for transport of solute to the particle phase (as the focus is on hydrodynamics, intra‐particle terms will not be treated in detail). A number of authors describe the non‐stationary particle phase by adding an extra equation for the solid phase concentration:^26^, ^27^, ^28^

(2) $$\varepsilon_{s}\frac{\partial q^{\prime }}{\partial t}=D_{s}\frac{\partial^{2}q^{\prime }}{\partial z^{2}}+S$$

where ε_s_ is the solids fraction, q′ is the average solute concentration in the solid phase and D_s_ is the solids dispersion coefficient. D_s_ is either determined experimentally^26^, ^29^, ^30^, ^31^, ^32^, ^33^, ^34^ or is calculated^27^, ^28^ via van der Meer's correlation:^35^

(3) $$D_{s}=0.04u_{f}^{1.8}$$

To account for varying particle size and density, algebraic expressions have been proposed, such as those by Tong et al. 27 (particle size over bed height), de Araújo Padilha and co‐workers^36^ (particle size and bed voidage) and Kaczmarski and Bellot^37^ (particle size and density over bed height). Wistrand and Lacki^38^ and Yun and colleagues^39^, ^40^ used an approach developed by Al‐Dibouni and Garside^41^ to model the combined effect of particle size and local hydrodynamics on the performance of an EBA column. Based on their simulations of a Streamline resin (GE Healthcare, Uppsala, SE) with different size and distributions, Wistrand and Lacki^38^ concluded that intra‐particle diffusion, liquid phase linear velocity and viscosity had important effects on column utilization, whereas axial dispersion and solids diffusion exerted negligible effects. Fennetau et al. 42 evaluated the effect of column diameter on the radial flow profile in 1 and 5 cm diameter columns. Their 2D simulations showed that the axial dispersion increases with column diameter due to the radial flow profile. However, their model assumed a constant velocity profile over the length of the column and did not take into account the effects of particle movement.

Various authors have put forward models that divide the column in several sections (not to be confused with spatial discretization in the numerical solution of partial differential equations (PDEs)). Typically these models are validated using multiple sampling points along the column height. Li et al. 43 developed a three‐zone model based on Wright and Glasser's axial dispersion model.^26^ Extensive research was performed by Yun and Lin,^39^, ^40^, ^44^, ^45^, ^46^ who used a multizone model with parameters based on regression of residence time distribution data. Lin et al. 46 showed that for resins with a wide size and density distribution, local hydrodynamics and HETP change considerably over the bed height.^47^

### Ideal, dynamic bed expansion

In the previous sections bed expansion was assumed to remain constant over time, and not influenced by changes in liquid velocity or viscosity. In reality these can have a considerable effect on the bed expansion. Thelen and Ramirez^47^, ^48^, ^49^ developed a rigorous model to allow for model‐based monitoring and control of expanded beds.^48^, ^49^, ^50^ Based on the conservation equations for two‐phase flow combined with the Richardson‐Zaki drag force expression they derived (4) for the void fraction.

(4) $$\frac{\partial \varepsilon_{s}}{\partial t}=-\frac{\partial }{\partial x}\left[\varepsilon_{s}u_{f}-\varepsilon_{f}D_{s}\frac{\partial \varepsilon_{s}}{\partial x}\right]$$

This equation can be applied to two extremes, zero dispersion and infinite dispersion. In the zero dispersion case, any changes at the inlet of the column (such as differences in liquid velocity, viscosity or density) will be advected with the liquid phase velocity and move along the column length in a sharp front, until they reach the top of the bed. In the infinite dispersion case, the particle phase is considered perfectly mixed and only the bed height reacts to changes at the inlet.

**Box insert 1. Technological developments of EBA resins and flow distributors**

The first EBA systems employed adsorbent particles from conventional packed bed systems. The low density of these particles (1.2–1.3 g cm^‐3^) limited the fluid velocity that could be used (typically 200–300 cm h^‐1^).^50^ To overcome the limitations this caused, particles with a densified core were developed, allowing linear velocities of up to 900 cm h^‐1^. Early densified resins incorporated glass or silica, while later metals such as steel or tungsten carbide were used.^51^ Several researchers have attempted to minimize unwanted interactions by modifying the adsorbent particle backbone. Examples include adding polymers^52^ or employing low temperature plasmas.^13^ While these modifications were found promising in academic research, they were not widely adopted by commercial resin manufacturers. An extensive overview of commercially available and custom made resins can be found in Li et al. 51

**Figure I.** Developments in EBA resin particle design.

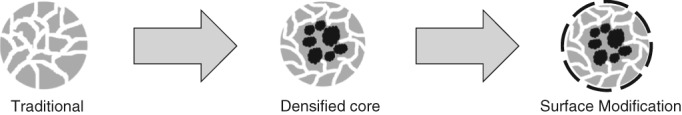

As is typical for packed bed columns, early EBA columns employed a perforated plate or mesh as flow distributor, sometimes in combinations with large, inert particles. Both designs were found to be very susceptible to fouling and difficult to clean, limiting their potential for use in the biopharma industry.^53^ Several designs were put forward that employed localized stirring in the bottom of the column.^54^, ^55^ One design, which used rotating/oscillating arms from which the fluid emerged had some commercial success,^56^ but suffered from the added mechanical complexity. Recently, a distributor design was reported that employed a crossflow mesh distributor.^57^ The inventors claim that the shear stress at the mesh surface prevents fouling, but no test results have been published to support this claim.

**Figure II.** Developments in flow distributor design for EBA columns.

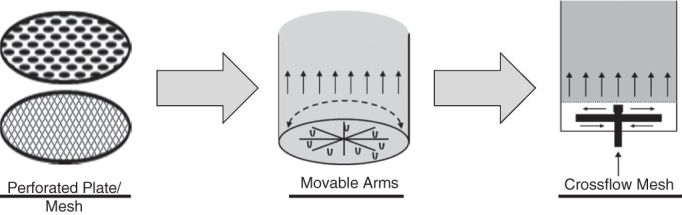

The two cases were validated using a lab scale EBA system with a Streamline resin (GE Healthcare, Uppsala, SE), which was subjected to step changes of inlet velocity and fluid viscosity (for bed expansion as well as bed contraction). The authors found the infinite dispersion model could successfully predict expansion and contraction profiles. However, as the particle size and density are averaged over the entire bed, this model would not allow for optimization of the particle size and density distributions. Nonetheless it appears the only attempt to describe bed height dynamics using multiphase fluid dynamics.

### Non‐ideal bed expansion

A major challenge in the operation of EBA in industry comes from the unwanted interaction of components such as biomass, DNA and lipids with the adsorbent particles.^59^ To investigate the effect of biomass on bed stability, the Villermaux and van Swaaij (VvS) extended PDE model^58^ (originally developed to describe non‐ideal flow patterns and stagnant zones in packed beds) has been used for EBA columns.^59^, ^60^, ^61^

**Figure 2:**
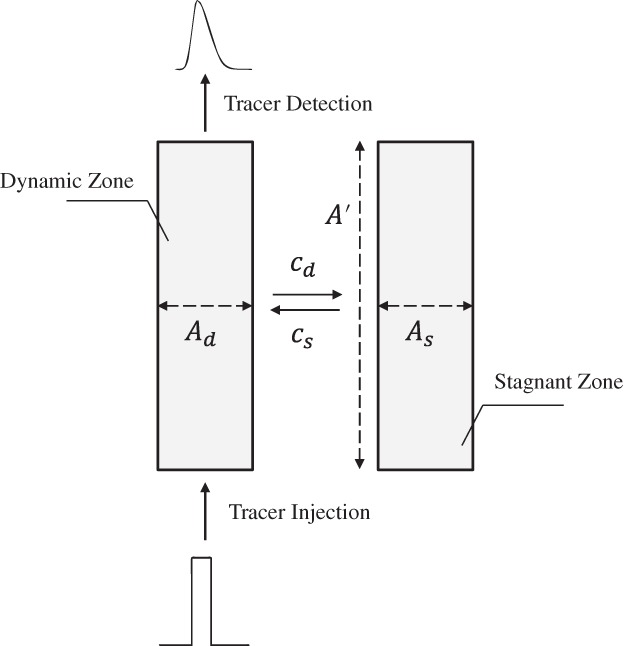
Schematic representation of the extended PDE model.

In this model the bed is divided into a perfectly classified (dynamic) and an aggregated (stagnant) region, as shown in Fig. 2. The transport of a solute in the dynamic and stagnant zone can then be described by two PDEs:

(5) $$\frac{\partial c_{d}}{\partial t}+u_{f}\frac{\partial c_{d}}{\partial x}=D_{f}\frac{\partial^{2}c_{d}}{\partial x^{2}}-\frac{{\mathrm{kA}}^{\prime }\;}{A_{d}}\left(c_{d}-c_{s}\right)$$

(6) $$\frac{\partial c_{s}}{\partial t}=\frac{{\mathrm{kA}}^{\prime }\;}{A_{s}}\left(c_{d}-c_{s}\right)$$

Here, *u*_*f*_ is the interstitial fluid velocity, *D*_*f*_ is the fluid phase dispersion coefficient, *c*_*d*_ and *c*_*s*_ are the tracer concentrations in the dynamic and stagnant phase, *k* is the mass transfer coefficient for transport between the dynamic and stagnant phase, *A*^′^ is the surface area between the phases and *A*_*d*_ and *A*_*s*_ are, respectively, the cross‐sectional areas of the dynamic and stagnant phases perpendicular to the flow direction. Using this approach Fernández‐Lahore *et al*.^59^ have indicated that if more than 20% of the bed becomes aggregated, the performance of the column is seriously affected because preferential flow paths (channelling) reduce the available surface area for adsorption. While this model is currently the only one capable of providing information on bed stability under more realistic process conditions, it does not indicate where in the column agglomeration is happening, nor what the local conditions were under which bed agglomeration took place.

## REVIEW OF MATHEMATICAL MODELS FOR LIQUID–SOLID FLUIDIZED BEDS

Outside of the context of DSP in bioprocessing, an expanded bed can be considered to be a classified, liquid–solid fluidized bed. Liquid–solid fluidized beds can be found in many fields such as biochemical, chemical, metallurgical and mineral processing industries.^62^ In the previous section on EBA models the solid phase has been described either by a 1D convection dispersion equation, or by assuming a perfectly classified bed with an analytical expression for the particle size, density and void fraction. While both approaches have been successfully used to describe early EBA systems, they require experimental data, for instance on the expansion factor. Also, any non‐ideal bed behaviour is described by only a few empirical parameters. In fluidized bed research fluid mechanics approaches are proven tools for predicting the complex behaviours of both the continuous (fluid) and dispersed (particle) phases.^63^ The following section aims to review the modelling techniques available in literature outside the field of EBA research with special focus on methods for incorporating polydispersity.

### Linear stability analysis

Early attempts to describe the stability of fluidized beds and the transition from the homogeneous to heterogeneous flow regimes were based on linear stability analysis (LSA) of a one‐dimensional set of mass and momentum equations. The concept of linear stability analysis is to superimpose infinitesimally small perturbations onto a variable at steady state and evaluate the transient behaviour resulting from it. Joshi *et al*.^64^ have applied the technique to a wide selection of multiphase systems, including LSFBs. An attractive side of this form of LSA is that it is based on the structure of the underlying equations, rather than its solution. This could prove beneficial to predict the bed stability of EBA systems subjected to challenging conditions. The majority of the LSA models have all assumed a constant particle size. In the field of sedimentation, which is governed by the same physics as a LSFB (only here the dispersed phase moves through a stagnant liquid phase), the linear stability model has been extended to include polydispersity.^65^ The extension of these approaches to fluidized systems could provide a good starting point to predict the stability of EBA systems.

### Multiphase computational fluid dynamics

The description of fluidization using multiphase fluid dynamics was pioneered by Anderson and Jackson.^66^ Starting from the Navier–Stokes equations and the equations describing the motion of a single particle, a set of continuum equations for the conservation of mass, momentum and energy of both the fluid and solid phase was derived (Box insert 2). To describe the behaviour of the solid phase, several methodologies exist. The most extensive and detailed options would be to resolve the flow and transport phenomena around each and every particle. This approach is known as direct numerical or fully resolved simulation. Due to the high grid requirements (on average around 1000 grid points per particle are required) this approach is currently limited to several thousands of particles.^67^ Another option is to set up and solve the flow variables using the volume averaged Navier–Stokes equations (VANS). The following section will evaluate the most widely used approaches and show how they can be used to model polydisperse systems.

### Eulerian computational fluid dynamics models

In the so‐called Euler–Euler (or two‐fluid) method for multiphase flow both the continuous and the dispersed phase are described using continuum equations. Both phases are considered to occupy the same volume space, each with their own volume fraction (Box insert 3). The momentum equations are coupled through the volume fraction and an inter‐phase coupling force. The main driver for the development of these models has been the simulation of gas–solid fluidized beds,^63^ but a number of authors has employed this method for LSFBs. An extensive overview can be found in Lettieri and Mazzei.^68^ One of the challenges when using a continuum description of the dispersed phase is the requirement for closure relationships for the particle pressure and viscosity. For a system having a single particle size this can be achieved using the kinetic theory for granular flow, proposed by Gidaspow.^69^ In an attempt to investigate the effects of polydispersity several authors have modelled bi‐disperse systems using two Eulerian solid phase descriptions for the solid phases.^70^, ^71^ As mentioned by Passalacqua and Fox^72^ this ‘multifluid’ approach can be extended to any number of phases. However, this comes at the cost of an extra set of mass and momentum equations for each solid phase. For 3D systems with one fluid and two solid phases this already results in a total of nine momentum equations (three sets of 3D equations). Furthermore, the strong inter‐phase coupling and particle stresses at higher particle fractions lead to a very stiff system of equations, which is difficult to solve with the algorithms found in most CFD software packages. To overcome this issue, the Eulerian model has been coupled to population balance solvers for the dispersed phase.^73^ This has proven successful for liquid–liquid,^74^ gas–liquid^75^ and gas–solid flows.^76^ To the best of our knowledge this method has not yet been successfully extended or validated for dense, polydisperse solid–liquid suspensions.

**Box insert 2. Solving computational fluid dynamics problems**

Computational fluid dynamics (CFD) consists of solving the transport equations for the conservation of mass, momentum and energy using numerical methods. While the specific implementation of the numerical methods may differ, all of them follow a similar sequence of steps, which is shown schematically in the figure below.

**Figure III.** Schematic overview of the sequence of steps in solving CFD problems.

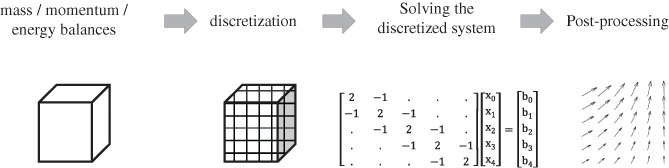

Simulating fluid flow starts by setting up the partial differential equations (PDEs) for the conservation of mass, momentum and energy over a domain. These system are typically coupled (variables are present in multiple equations) and can be stiff in nature (exhibit dynamics of multiple timescales). To create a solvable system a set of boundary conditions is required plus (or transient simulations) an initial condition. Discretization involves dividing the spatial domain up in smaller elements or cells (also referred to as a mesh). This converts the PDEs into a system of coupled ordinary differential equations (ODEs). For each time step this results in a matrix equation which has to be solved numerically. The (sparse) matrices emerging from the discretization procedure have to be solved (repeatedly for transient simulations). For small 1D/2D problems this can be done using a so‐called direct solver (which uses a form of Gaussian elimination). This method requires a large amount of computer memory so for larger (3D) problems iterative linear solvers are required. During the simulation the variables that are solved for are saved to disk. The data can then be post‐processed to yields plots and figures of flow fields, etc.

### Lagrangian description of dispersed phase

In the Euler–Lagrange approach the continuous phase is still described by continuum mass, momentum and energy equations, while the dispersed phase is considered as a set of discrete particles (box insert 3). This discrete treatment of the solid phase removes the difficulties with polydispersity found in continuum models. The motion of each particle is calculated using Newton's second law which states the acceleration of an object is equal to the sum of the forces acting on it, divided by its mass. Several ‘flavours’ of the Lagrangian formulation exist, which differ in the way the inter‐particle collisions are modelled. Two widely used methods are CFD combined with the discrete element method (DEM) and the multiphase particle‐in‐cell method (MP‐PIC). In DEM, particle interactions are modelled by considering the particles to be small, soft spheres (box insert 3). The method was originally proposed by Cundall and Strack^77^ for granular assemblies, and later applied to fluidized beds by Tsuji *et al*.^78^ An extensive review of the CFD‐DEM can be found in Zhou *et al*.^79^ The CFD‐DEM method has been used by Peng *et al*.^80^ to investigate the effect of segregation and solid dispersion in a bi‐disperse LSFB. Their simulations were found to be in good agreement with experimental data. Since the local properties of both the liquid flow and particle motion were available from the simulation, the effects of void fraction, liquid velocity and particle properties on the solids dispersion coefficient could be evaluated in detail.

### The multiphase particle‐in‐cell method

The MP‐PIC method can be thought of as a hybrid method that aims to combine the Lagrangian description of the dispersed phase with the computational efficiency of a two‐fluid solver. The main difference between the DEM method described in the previous section and MP‐PIC is that the latter does not resolve collisions between particles. Particle–particle collisions are handled using a stochastic model based on a ‘particle pressure field’ and the fluid and solids relaxation timescale models.^81^ All currently used MP‐PIC models rely on grouping particles in computational parcels. The use of a stochastic collision model requires the amount of particles to be high enough for statistical averaging. As this can mean that hundreds of particles have to be grouped together, this method is not well suited for the prediction of local particle properties, such as solids dispersion coefficients. Until recently the MP‐PIC model has been primarily validated in gas–solid systems. In LSFBs, the coupling between the phases is stiffer, as the densities are closer in value.^82^ A recent comparison of Lagrangian models for LSFBs showed that the MP‐PIC method showed only minor computational speed‐ups at the expense of considerable loss in accuracy.^83^

**Box insert 3. Methods for solving multiphase CFD problems**

**Eulerian–Lagrangian**

To compute fluid flow, mass and heat transfer in multiphase systems a variety of methods can be employed. The (conceptually) least complex approach is known as fully resolved CFD‐DEM or direct numerical simulation (DNS). Here, the mesh is smaller than the particle diameter and all the flow details around the particles are computed (Fig. IV). The particle surface is seen as a solid boundary for the fluid and particle movement is computed by evaluating the forces in it. A large number of grid cells are required. To compute all the flow paths, typically in the order of 1000–10 000 cells per particle. This limits the DNS approach to small systems of around 1000–10 000 cells simulated over time ranges in the order of seconds.

**Figure IV.** Examples of meshes usedin resolved (left) and unresolved (right)Eulerian–Lagrangian simulations.

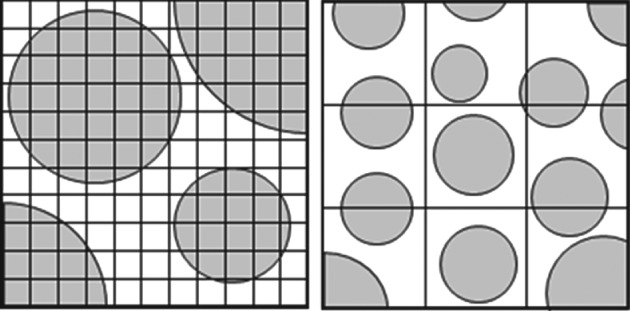

**Figure V.** Transformation of discreteparticles to a continuum descriptionusing void fractions.

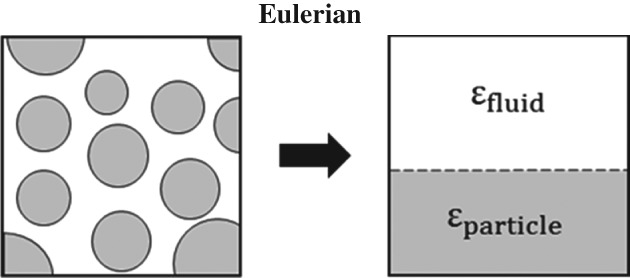

**Figure VI.** Schematic overview of the spring/dash‐pot slider model.

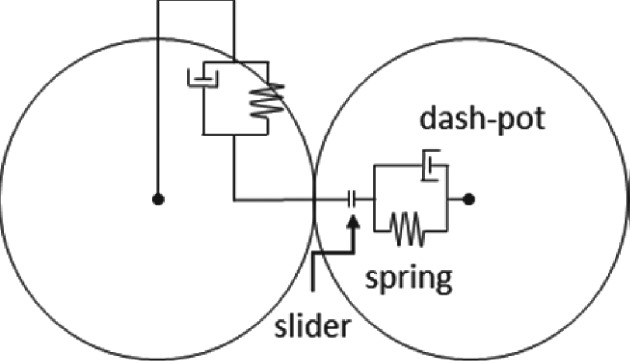

The unresolved CFD‐DEM method uses bigger grid cells than the DNS method (Fig. IV). Typically several particle diameters are used as cell size. The governing PDEs are extended to include the particle volume fraction and source terms for exchange of mass, momentum and energy. These are then interpolated between the grid cells and the particles. Since not all details of the local flow field are computed, semi‐empirical correlations are required to describe transfer at the fluid/particle interface. This method allows for larger systems of over a million particles to be simulated over time range in the order of minutes. The predictive quality of the simulations does heavily depend on the used correlations for particles/fluid forces, which is currently an active field of research.

In the Eulerian description (also referred to as two‐fluid or multi‐fluid), the dispersed phase is not seen as discrete particles. Instead both phases are seen as inter‐penetrating continua, represented by a volume fraction (shown as *ϵ*_*fluid*_ and *ϵ*_*particle*_ in Fig. V.

Since all phases are now represented by PDEs, the computational requirements of this model are dictated by the number of grid cells (i.e. the desired resolution of the simulation). This allows large‐scale industrial equipment to be simulated over long timescales. As with the CFD‐DEM method, semi‐empirical correlations are required to describe interaction and exchange between the phases.

For a mono‐dispersed system, the number of particles and the surface area can be directly calculated from the volume fraction. This cannot be done for poly‐dispersed systems. This limits the use of this method in situations that display clear gradients or segregation in particle sizes.

**Discrete element method**

In the discrete element method (DEM) solid particles are tracked using Newton's laws of motion (acceleration = sum of forces divided by the mass). The tracking of individual particles naturally allows for individual particle properties such as size and density. To model the collision between solid particles, the particles are treated as soft spheres. When two particles make contact, the collision forces use a combination of a spring, dashpot and slider. The combination of the spring stiffness, the viscous dampening of the dash‐pot and the friction value of the slider can be set to match the material properties of the particles (Fig. VI). However, the small time steps required to accurately resolve the make this method very computationally expensive.

## CASE STUDY: OPTIMIZING THE DESIGN OF A ROTATING FLUID DISTRIBUTOR USING CFD

For proper application of the incoming feed stream to an EBA column, various designs for fluid distributors have been put forward. While differing in shape and mode of operation, the general aim of these devices is to provide a homogenous liquid and prevent/avoid fouling and clogging. As mentioned in box insert 1, EBA columns were developed that employed fluid distributors consisting of several radially oriented arms which rotate/oscillate at the bottom of the column. The rationale behind this design was that the distributor motion prevents any dead zones in the bottom of the column, while still providing a fully cleanable flow path.^54^, ^55^, ^86^ Arpanaei *et al*.^54^ evaluated and compared this design for several column diameters, liquid velocities and rotation/oscillation frequencies. Based on experimental measurements of an inert tracer the HETP and Bodenstein numbers were calculated. However, the placement and orientation of the holes through which the liquid exited the arms was not investigated.

**Figure 3:**
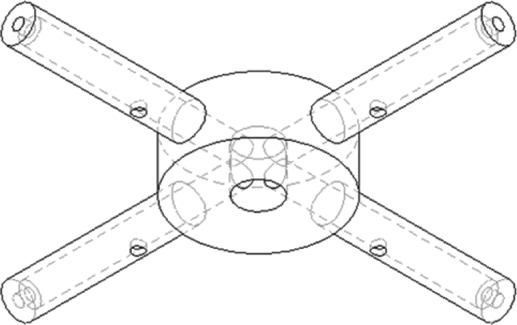
Base case design with a hole on the tip and a hole on the bottom of the distributor arm.

**Figure 4:**
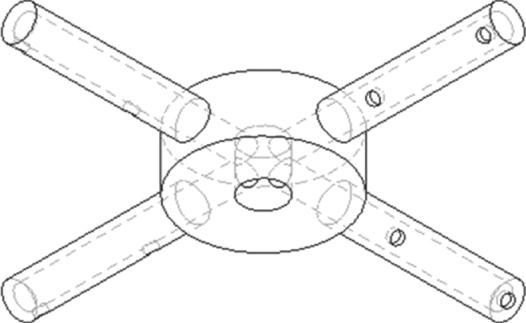
Improved distributor design with two backwards angled holes, both located on the side of the distributor arm.

To investigate this effect in more detail, a single phase CFD model was constructed using Ansys Fluent 15 (Canonsburg, PA, USA). The flow field (velocity and pressure) was computed using a steady state solver. Using this flow field, the motion of an inert tracer was simulated over the course of 60 s. The simulation domain consisted of a 10 cm column, in which a distributor with four arms was placed. Two distributor designs were compared, which are shown in Figs 3 and 4. The base case design (based on patent and literature data^54^, ^84^) had two orifices, one pointing downwards and one pointing towards the column wall. The improved design also had two orifices, both angled 45^o^ in the direction away from the rotating direction of the distributor arm.

**Figure 5:**
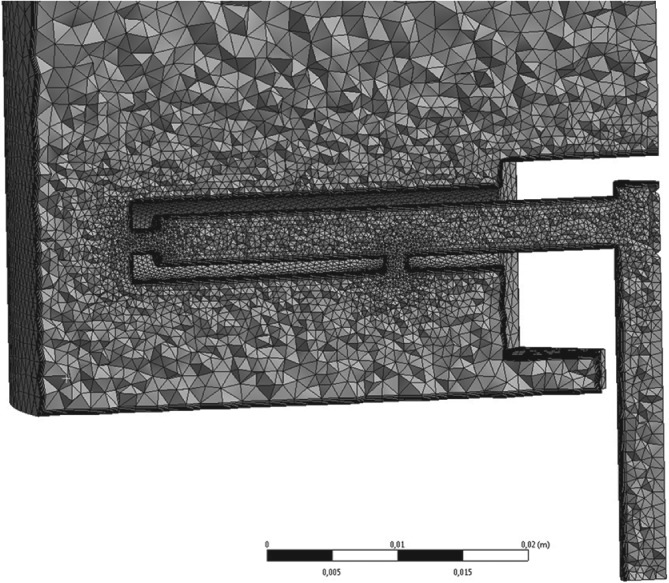
Bottom section of the mesh used for the base case distributor design, sliced along the YZ plane, viewed from the negative x‐axis.

The meshes of the base case and the improved design consisted of 701296 and 711590 elements, respectively. An unstructured mesh was used which was refined near the distributor arm and inlet orifices (this is shown for the base case in Fig. 5). Three fluid velocities (300, 500, 750 cm h^‐1^) and three rotation speeds (5, 10 and 15 rpm) were tested. The fluid properties used were those of water (taken from the Fluent material database). For the tracer studies acetone was used (taken from the Fluent material database), with a mass fraction of 0.01. This mass fraction was deemed low enough to assume a constant bulk density, thereby making the tracer fraction a passive scalar.

**Figure 6:**
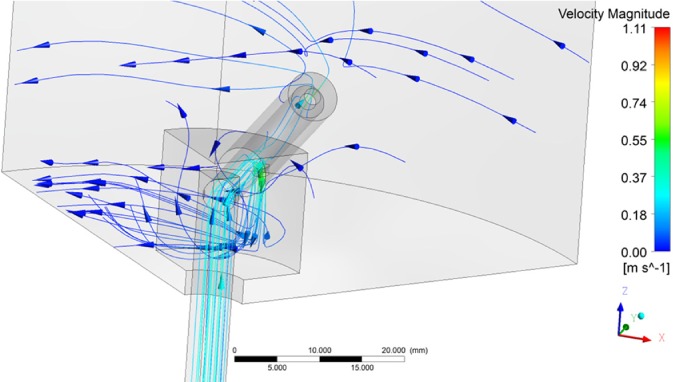
Flow field for the base case distributor design (linear column velocity: 500 cm h^‐1^, rotation speed 5 rpm).

**Figure 7:**
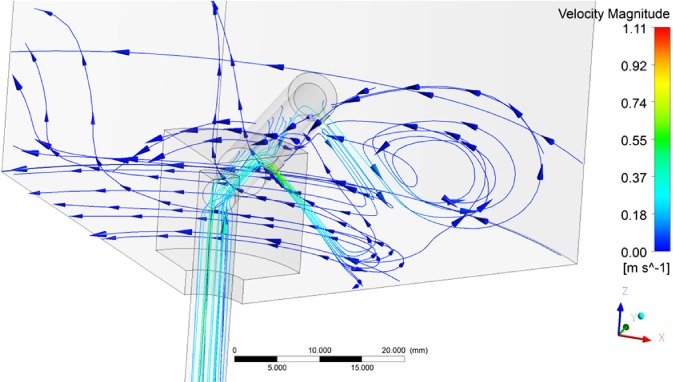
Flow field for the improved distributor design (linear column velocity: 500 cm h^‐1^, rotation speed 5 rpm).

The two tested distributor designs behaved similarly under varying liquid velocities but showed different behaviour when the rotation speed was varied. Visualizations of the flow fields are shown in Figs 6 and 7. In both cases the general solid body rotation caused by the distributor arms can be observed. In the improved design a recirculation pattern can be observed at the rear of the arm.

**Figure 8:**
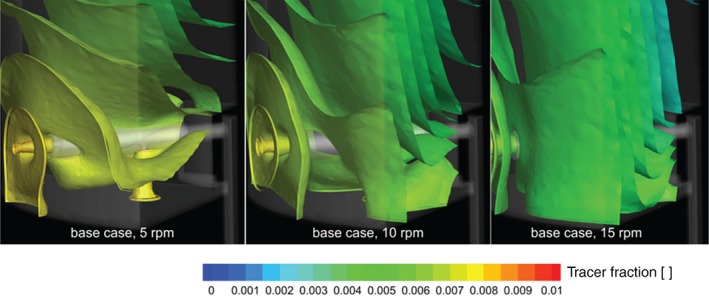
Contour plots of tracer fraction after 60 s (normalized by incoming tracer fraction) for the base case design at different rotation velocities (all with a linear column velocity of 500 cm h^‐1^).

**Figure 9:**
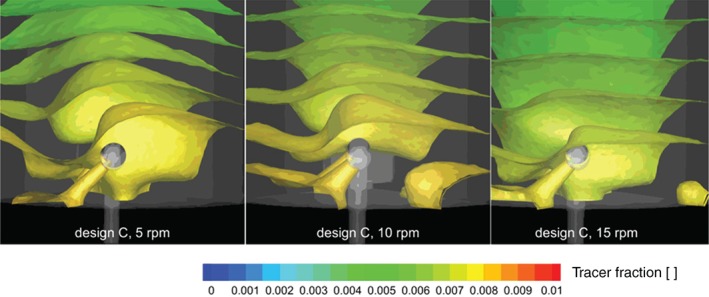
Contour plots of tracer fraction after 60 s (normalized by incoming tracer fraction) for the improved design at different rotation velocities (all with a linear column velocity of 500 cm h^‐1^).

In Figs 8 and 9, snapshots of the tracer motion are shown through contour plots. The centrifugal effects in the flow field, caused by rotation of the arm are visible in the curved tracer contours of the 10 and 15 rpm snapshots of the base case design.

The tracer plots in Fig. 9 indicate the recirculations, caused by the interactions of the jets exiting the arm with the bulk rotation flow, reduce the centrifugal effects in the flow field. As a result, the tracer contours have a ‘flatter’ horizontal profile, indicating a more plug flow like flow regime in the column.

**Figure 10:**
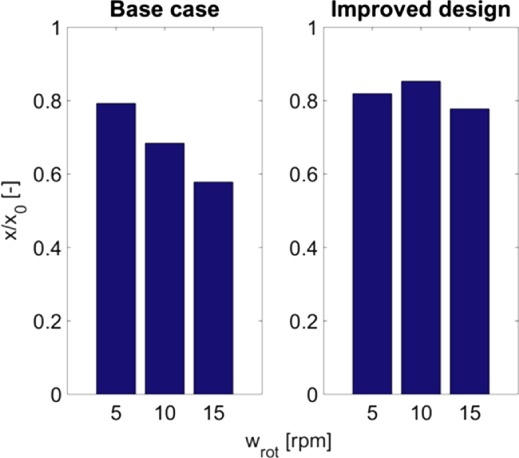
Mean normalized tracer fractions after 60 s at a plane height of 2 cm for different rotation speeds (all with a linear column velocity of 500 cm h^‐1^).

**Figure 11:**
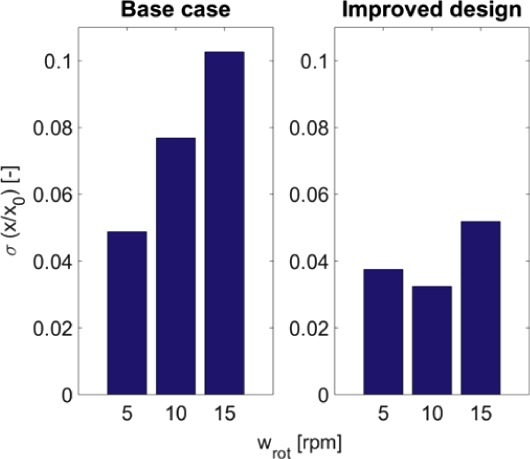
Standard deviations of tracer fraction after 60 s at a plane height of 2 cm for different rotation speeds (all with a linear column velocity of 500 cm h^‐1^).

To allow for a quantitative comparison of two designs, the average and standard deviation of the tracer fractions were evaluated at a horizontal plane at 2 cm height (just above the fluid distributor). The results are shown in Figs 10 and 11, again for varying rotation speeds. It can be observed that for the improved design at all rotation speeds, the tracer fraction shows a higher average and lower standard deviation. This further confirms the ‘flatter’ profile indicated in Fig. 9. Furthermore, a rotational speed of 10 rpm appears to be optimum (in terms of high average and low standard deviation).

This illustrates how CFD not only provides a ‘visual aid’ for design exploration, but also allows optimization of operating conditions. Furthermore, knowledge on the dispersion in the distributor region of the column can improve how well simplified models (such as those used in flow sheeting software) approximate industrial scale equipment. This allows engineers to optimize operations at an early stage of the process design, reducing the time and effort required to bring process designs to full scale operation.

## CURRENT CHALLENGES AND POTENTIAL IMPROVEMENTS

Since the introduction of EBA, a variety of model equations has been proposed to describe its operation. A summary overview of these models, is given in Table 1.

**Table 1:** Schematic overview of models presented in the paper

Model type	Capabilities	Drawbacks	Most suitable application
HETP	Quick solutions without the need for numerical methods	Solid phase does not behave as a series of tanks	Back of the envelope calculations for column sizing
Convection–dispersion	Species transport, mass transfer and adsorption over multiple column runs	Does not include bed dynamics	Optimization of column size and process conditions
Stokesian two‐phase flow	*A priori* prediction of bed height dynamics	Constant particle size limits use for solid phase optimization	Monitoring/control (predicting of bed height after changes in fluid flow rate of liquid phase composition
Extended PDE (Villermaux van Swaaij)	Indicates solid phase agglomeration using online measurements	No information of location of agglomeration, time delay in information, no predictive capabilities	Monitoring/control (detecting solid phase agglomeration)

Currently no model is available that is able to address bed expansion, mass transfer, adsorption, and unwanted interactions that lead to bed agglomeration. To allow process and equipment design for EBA systems, it is vital that the dynamic bed behaviour can be predicted while operating under realistic conditions. This means current models will have to be improved or extended. In multiphase reactor engineering, the use of multiphase computational fluid dynamics has been widely adopted,^85^ which has improved fundamental knowledge on the stability and optimal operation of fluidized beds. CFD studies have already proven successful in PBC and EBA flow distributor design.^57^, ^86^ However, one major drawback of the multiphase CFD methods (both CFD and DEM) is the limited time that can be simulated. Even with state of the art algorithms on modern supercomputers, the maximum time that can be simulated is in the order of minutes.^87^ As a consequence, simplified (reduced order) models will still be required to describe a separation process consisting of multiple consecutive equilibration, load, wash, elution, regeneration/cleaning steps.

**Figure 12:**
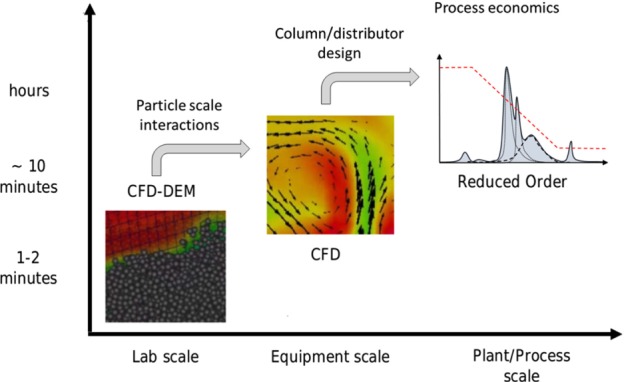
Overview of a multi‐scale simulation cascade for simulating EBA systems.

For successful implementation of EBA for industrial separations, the benefits of different simulation strategies should be combined. This is shown schematically in Fig. 12. CFD‐DEM simulations can be used to obtain particle scale data such as particle dispersioncoefficients, mass transfer coefficients and particle agglomeration behaviour. The physics of agglomeration are similar to solid phase flocculation.^88^ Previous work in this field, such as Thomas *et al*.^89^ or Bridgeman and co‐workers^90^ could provide good starting points for modelling agglomeration in EBA columns. Due to the Lagrangian description of the particle phase, particle size and density distributions can be easily incorporated. Using Eulerian‐CFD models column and distributor design can be optimized. This improves the desired plug flow through the bed and ensures no ‘dead zones’ interfere with cleanability. Finally, reduced order models can be employed in flow sheeting (the use of computer programs to solve the mass and energy balances of the process to calculate equipment sizes and process economics). By comparing optimized EBA with alternative unit operations, more quantified decisions can be made at an early stage of process development. This reduces risks and delays, and assures EBA is used in situations where it's potential can be fully employed.

## SUMMARY AND CONCLUSIONS

To successfully apply EBA in an industrial setting, model based optimization of resin, equipment and process conditions is required. For this, a variety of models for EBA has been proposed in the literature, but the combined simulation of column geometry, dynamic bed expansion, mass transfer, adsorption and agglomeration has not been addressed. In multiphase reactor engineering, the use of multiphase computational fluid dynamics has been shown to improve fundamental understanding fluidized beds. To advance EBA technology, a combination of particle, equipment and process scale models should be used. By employing a cascade of multiscale simulations, the various challenges EBA currently faces can be addressed. This allows for optimal selection of equipment, materials and process conditions and reduces the risk and development times of DSP involving EBA.
